# Presence of SARS-CoV-2 on food surfaces and public space surfaces in three districts of Lima, Peru

**DOI:** 10.1590/1414-431X2022e12003

**Published:** 2022-07-13

**Authors:** K. Alvis-Chirinos, Y. Angulo-Bazán, O. Escalante-Maldonado, D. Fuentes, M.G. Palomino-Rodriguez, E. Gonzales-Achuy, H. Mormontoy, P. Hinojosa-Mamani, L. Huamán-Espino, J.P. Aparco

**Affiliations:** 1 Centro Nacional de Alimentación y Nutrición, Instituto Nacional de Salud, Lima, Perú; 2 Oficina General de Investigación y Transferencia Tecnológica, Instituto Nacional de Salud, Lima, Perú; 3 Centro Nacional de Salud Pública, Instituto Nacional de Salud, Lima, Perú; 4 Departamento Académico de Nutrición, Universidad Nacional Mayor de San Marcos, Lima, Perú

**Keywords:** Inert surfaces, Food, SARS-CoV-2

## Abstract

The aim of this study was to determine the presence of SARS-CoV-2 on food surfaces and surfaces in public spaces in 3 districts of Lima, Peru. A cross-sectional descriptive study was carried out in three districts of the Lima metropolitan area. Surfaces that were most exposed to users were selected. Samples were swabbed for 4 weeks and transported to the laboratory to determine the presence of the virus. One thousand ninety-five inert surface samples and 960 food surface samples were evaluated for the identification of SARS-CoV-2 by the real time-PCR molecular test, whereby only one sample from an automated teller machine was positive. Most of the inert and food surfaces evaluated did not show the presence of SARS-CoV-2 during the time of sample collection. Despite the negative results, the frequency of disinfection and hygiene measures on high-contact surfaces should be maintained and increased to prevent other highly contagious infectious diseases.

## Introduction

An epidemic outbreak of pneumonia of unknown origin began in Wuhan-China in December 2019 and quickly spread throughout the world. The World Health Organization (WHO) named this disease COVID-19 and declared it a global pandemic in March 2020. After one year of pandemic, around 123 million confirmed cases and 2 million deaths have been reported (1), in addition to the important socio-economic problems.

In Peru, the first case of COVID-19 was identified on March 6, 2020, and the first two deaths occurred thirteen days later. The highest number of deaths per day occurred in July (590.4 average deaths per day), and the highest number of confirmed COVID-19 cases occurred in August (7,847.1 average cases per day). However, an important decrease in deaths and cases was observed in November, 103.5 deaths per day and 1,663.6 cases per day (2). As of March 2021, there were about a 1.5 million confirmed cases and 50,000 deaths. The Peruvian government has decreed a continuous national state of emergency since March 2020 to the present. The right to freedom of movement and productive activities were restricted in our country and social isolation has been emphasized in order to reduce the transmission of SARS-CoV-2 (3).

COVID-19 is caused by SARS-CoV-2, an RNA virus belonging to the Orthocoronavirinae family, which also includes other agents that caused epidemics such as the Middle East Respiratory Syndrome or Severe Acute Respiratory Syndrome (SARS-CoV) (1). One of the most important characteristics of SARS-CoV-2 is its transmissibility, with mean basic reproduction number (R0), defined as the average number of new cases generated by a case (4), of 2.2, but which can range from 1.4 to 6.5 (5). This not only justifies measures of social isolation, but also requires research on other possible forms of transmission that do not involve close contact between people.

The WHO has suggested that the transmission of this virus can occur indirectly by contact with surfaces in the immediate environment or with objects used by the infected person (6). Various studies confirm that SARS-CoV-2 can remain on different types of materials from two hours to nine days (7). Although it is considered that respiratory droplets or aerosols from an infected person are the main form of contagion, it is postulated that the virus could remain active on surfaces and that this route would cause infection by direct contact with infected objects (8). In this regard, the WHO states that infected droplets are too heavy to be transported through the air and land on objects (including food) and surfaces that surround the infected person (9). Thus, viable SARS-CoV-2 virus and/or RNA detected by RT-PCR can be found on these surfaces for periods ranging from hours to days, depending on the environment (including temperature and humidity) and the type of surface.

Previous studies have reported virus transmission through food, when the mode of spread is direct (10). However, the European Food Safety Agency (EFSA) as well as the WHO have declared that food are not agents of transmission of SARS-CoV-2 (11). Nonetheless, the EFSA claim is based on studies related to viruses such as SARS-CoV-1. So far, studies using SARS-CoV-2 have not been conducted. It is important to mention that in Europe food production and distribution is industrialized, where food processing is mechanized, reducing the opportunity for human intervention. In our country, food handling by people is direct and frequent. Most of the food in the markets is not packaged and is touched by the seller and buyer. Some studies in Peru (12) mention that this represents a risk for disease transmission.

There is little information on the actual viral load that can be found on inanimate surfaces and food surfaces, especially those that are frequently touched by people (13). That is why the WHO already recommends ensuring adequate disinfection and cleaning of these surfaces. Most of the studies that have addressed the coronavirus burden have been conducted under laboratory conditions and have not included inert surfaces in the community or food. Therefore, the objective of the study was to determine the presence of SARS-CoV-2 on food surfaces and other surfaces of public spaces in districts of Lima, Peru.

## Material and Methods

### Study design

The study was observational, cross-sectional, and descriptive.

### Population and sample

The population consisted of food surfaces and inert surfaces in the districts of San Martin de Porres, Villa el Salvador, and San Juan de Lurigancho in Lima, Peru. The food samples were collected at local markets. The inert surfaces were collected at local markets, supermarkets, banking agencies, and the urban transportation system.

The districts of San Juan de Lurigancho, San Martin de Porres, and Villa el Salvador were selected because they were the districts with the highest number of COVID-19 cases in Lima as of May 1, 2020, according to the report of the National Center for Epidemiology, Prevention and Control of Diseases of the Ministry of Health.

In each district, the busiest markets, supermarkets, and banking agencies were selected, as well as the public transportation lines that travel the longest distance through the districts. Non-probabilistic convenience sampling was used to select the samples of food and inert surfaces. Four samples (two food samples and two control samples) of each food were collected for the determination of SARS-CoV-2 on surfaces. The foods included bulk rice, avocado, banana, mango, lemon, tomato, lettuce, potato, cheese, and chicken. For inert surfaces, five samples were taken for each commercial establishment (supermarkets, markets, and banking agencies), and another five control samples. Ten samples were collected in each transport unit (three on handrails, six on seats, and one on the door). Samples were collected once a week and four times per month from November to December 2020 as shown in Supplementary Table S1.

### Data collection

Data collection was carried out by technical laboratory personnel, trained in the collection, conservation, and transport of samples to the Peruvian National Institute of Health (INS). Prior to sample collection, authorization for the entry of field personnel was coordinated with those responsible for markets, supermarkets, banking agencies, and transport units in each district. For the food samples, a regular purchasing procedure was carried out, so the technical staff went to the stalls, selected and bought the food, and transported it to the laboratory. For inert surfaces, the technical staff in coordination with the person in charge of the commercial establishment indicated the surfaces to be sampled. The sample collection was carried out using the Jun Nuo Viral Transport Medium (VTM) (Shandong Chengwu Medical Products, China). The swabs were moistened in the VTM and passed on the food surfaces or inert surfaces. The swabs were immediately placed in the VTM, identified, and kept at 4°C (14,15) during transportation to the National Reference Laboratory for Respiratory Viruses of the INS, where the samples were processed within 48 h.

### Molecular diagnosis of SARS-CoV-2

The Zybio Nucleic Acid Detection Kit (Zybio Inc., China) for SARS-CoV-2 - Magnetic Bead Method (16,17) was used for the qualitative detection of RNA from swab samples. Briefly, the samples were homogenized and 200 µL was added to the 96-well extraction plate containing the extraction reagent (lysis buffer). Then, 15 µL of proteinase K was added and the plates were sealed with Parafilm^®^ M Laboratory Film (Bemis Company, Inc., USA). RNA extraction was performed using the EMX-6000 equipment (Zybio Inc.), where the extraction reagent I, magnetic bead kit, extraction reagent II, and elution reagent plates were placed following the instructions. The magnetic rack cover (blanket) was placed on the magnetic bead plate and 200 µL of nuclease-free water was added to the extraction reagent I plate (extraction control). For amplification, 5 µL of purified RNA was transferred to the amplification plate from position A1 to E12 that included 91 samples, the extraction control and aliquot control, 5 µL of the negative control, and 5 µL of SARS-CoV-2 RNA characterized by sequencing as positive control. The amplification plate was sealed and placed in the SANSURE MA6000 thermal cycler (Sansure Biotech Inc., China), using the fluorophores FAM (Gen *N*), ROX (Gen *ORF1ab*), VIC (internal control). According to the instructions of this qualitative kit, cycle threshold (Ct) greater than 39 for the ORF1ab gene is considered negative for the presence of molecular detection of the SARS-CoV-2 genetic material.

### SARS-CoV-2 isolation

The samples with a positive result for SARS-CoV-2 were homogenized and filtered for sterile condition. For SARS-CoV-2 isolation, the VERO 81 cell line (ATCC^®^ CCL 81 TM, USA) originated in African green monkey kidney was used. VERO 81 cells were cultured in DMEM (Dulbecco's modified Eagles medium) culture medium supplemented with streptomycin 100 mg/L, ampicillin 25 mg/L, 20% inactivated fetal bovine serum (SFB), and kept at 37°C in a humid atmosphere of 5% CO_2_. The filtered sample was inoculated into the cells and incubated for 7-10 days for the isolation of the SARS-CoV-2 virus. The presence of cytopathic effect was observed and the supernatant and cells were collected at the end of incubation. The isolation was determined by RT-PCR values.

### Study variables and indicators

The variables analyzed were presence of SARS-CoV-2 (laboratory determination, presence or absence), isolation (positive or negative), district [San Juan de Lurigancho (SJL), San Martin de Porres (SMP), and Villa el Salvador (VES)], food surface (bulk rice, avocado, banana, mango, lemon, tomato, lettuce, potato, cheese, and chicken), inert surface (handrail, seat, counters, touch screen, shopping cart handle, vending stand, and ATMs), sample origin (market, supermarket, public bus, train, and banking agency).

### Analysis of data

The information was registered on data sheets and the results of the molecular tests were entered into an Excel database. The relative and absolute frequencies of the main variable and secondary variables were determined. A univariate analysis was performed, obtaining frequency distributions for the qualitative variables. The analysis was carried out in the statistical program SPSS V25 (IBM, USA).

### Ethical aspects

The study protocol was reviewed and approved by the INS Institutional Research Ethics Committee (code OI-034-20).

## Results

Two thousand and fifty-five samples (960 food surfaces and 1095 inert surfaces) were collected and analyzed in 3 districts of Lima, Peru. No food surface sample was positive for SARS-CoV-2, while the virus was identified only in one of the samples from ATMs from the San Martín de Porres district, which represented 1.4% of the samples collected in this type of surface (Supplementary Table S2). The inert surface positive for SARS-CoV-2 had a Ct value of 35.51 for ORF1ab genes and no Ct for the N gene.

Subsequently, the SARS-CoV-2 positive sample was inoculated into a VERO 81 cell culture for 10 days for virus isolation. The supernatant and cells collected for virus isolation and the RT-PCR results showed a Ct value of 37.08 for the ORF1ab gene and no Ct for the N gene, indicating a low viral load and unreplicated virus, with the RNA likely being the remnants of the inoculum from the original sample. Additionally, no cytopathic effect was observed in the culture. The subcultivation of the isolated virus did not increase viral load, and finally the sample was found to be negative for SARS-CoV-2. Ct values are shown in Supplementary Table S3.

## Discussion

The present study aimed to determine the presence of SARS-CoV-2 in food and inert surfaces of markets, supermarkets, banks, and means of transportation in 3 districts of Lima, Peru. The results showed that SARS-CoV-2 was found only on the inert surface of one ATM machine.

As far as we investigated, this is one of the first reports of the presence and stability of SARS-CoV-2 in fresh food (without refrigeration) and under real conditions (outside the laboratory). Therefore, the results cannot be directly compared with the available literature. Yépiz-Gómez et al. (18) studied the recovery and survival of two respiratory viruses, an adenovirus (HAdV-2) and a coronavirus other than SARS-CoV-2 (HCoV-229E), in lettuce, strawberries, and raspberries stored at 4°C (refrigeration), and reported that the coronavirus was able to recover in the first 3 days with an efficiency of up to 19.6%. Dai et al. (19), evaluated the survival of SARS-CoV-2 in salmon artificially infected with SARS-CoV-2 inoculum, dried with filter paper, and stored at room temperature (25°C) and refrigeration (4°C) and found that the meat stored at room temperature showed viable virus for up to two days, while in the refrigerated sample the virus survived for 8 days.

The reasons why we did not find SARS-CoV-2 in the analyzed food samples could be due in part to the fact that the samples were collected during the period of greatest decrease in COVID-19 infections in Peru. The reduction in cases and deaths per day in 2020 occurred between August and November, from 7,847 to 1,663 cases and from 563 to 103 deaths per day. Specifically, during the sample collection period, there was an average of 1,575 cases and 103 deaths per day (2). Secondly, the foods that were purchased in markets and supermarkets could have been exposed to cleaning substances such as alcohol, bleach, etc, due to the biosafety measures implemented (20,21) that involved the sterilization of spaces and the use of disinfectants, which could reduce the number of viral particles. However, it should be noted that the collection of samples was carried out during times of high traffic of people and, in some cases (food and ATMs), this was unexpected. In addition, unlike previous studies (19,22,23) that found SARS-CoV-2 in food, in the present study the food was not artificially infected. We aimed to assess the contamination of food by manipulation and interaction of people in the markets. Also, the food was kept in the markets and transported to the laboratory at room temperature (17°C). Our results on fresh food surfaces are consistent with the EFSA and Food and Drug Agency statement that there is no evidence of transmission of COVID-19 through food (11,24). Likewise, the International Commission on Microbiological Specifications for Foods has prepared a joint declaration of the low risk of SARS-CoV-2 transmission by food (25).

Additionally, in the case of inert surfaces, SARS-CoV-2 RNA was only found in one sample (0.06%). The evidence is consistent with that of previous studies that used the same methodology on inert surfaces of hospital wards (26-28), in which no positive results were found. Other studies, however, evaluated the presence of SARS-CoV-2 genetic material on other high-traffic surfaces such as buses and trains (29), garbage can handles, in entries and exits of essential shops (30), and parks and water sources (31). These studies did find positive results, which ranged from 4-8%.

The relative contradiction between these results can be explained by some factors. For example, Kwon et al. (32) postulate that environmental temperature plays an important role in the stability of this virus, making it more difficult to detect as temperature increases, such as in summer. Likewise, the study by Eslami and Jalili (33) suggests that the relative humidity of the environment could affect the stability of the virus since in areas with higher relative humidity there were fewer cases of COVID-19. In our study, the collection of the samples took place between the months of November and December, when the temperature in the city of Lima is 17.7°C on average, with 84.2% relative humidity (34). This could explain the absence of viable viral particles. Moreover, the material in which the virus is found can influence its detection: its average duration in plastic and steel, the materials of most of the inert surfaces evaluated, is between three and four days (8).

It should be mentioned that some authors (14,35) have evaluated the ability to detect a predetermined SARS-CoV-2 viral load on surfaces similar to those in our study, and they mentioned that environmental conditions (temperature and relative humidity) could affect the infectivity of the virus: at higher temperature or relative humidity the viral load decreases.

Furthermore, Adem et al. (36) reported that the stability of the virus in plant foods can be affected by the phenolic substances of the vegetable peel, which inhibit the main protease of SARS-CoV-2. Nevertheless, there is a risk that the use of wastewater contaminated with feces from infected patients in the irrigation of vegetables can transmit the virus (37). In foods of animal origin, substances such as heparin and heparan (38), which are present in beef, poultry, pork, and seafood, are compounds that facilitate binding of SARS-CoV-2 to target cells (39), so there is a risk of spreading the virus through meat.

The strengths of the study are a robust sampling design that covers the geographical and temporal dimensions, which allowed the collection of samples in 3 densely populated districts, during 4 consecutive weeks and 4 repetitions of each sample of 10 types of food and 13 types of inert surfaces, totaling 2016 samples. Likewise, the samples were transferred fulfilling the technical criteria, and the collection of the samples in the field and the laboratory analysis was carried out by personnel trained and experienced in respiratory viruses. At the beginning of the pandemic in 2020, ZyBio Inc, a Chinese company, developed a new coronavirus nucleic acid detection kit (PCR fluorescent probe method) and nucleic acid extraction kit (magnetic bead method), and both successfully received EU CE certification in March, 2020 (16). Additionally, both methods obtained registration and emergency approval certificates from the National Medical Products Administration to be used throughout China. In addition, unlike previous studies conducted in the laboratory using spiked samples, our study was conducted under real-world conditions aiming to assess alternative routes of infection, rather than inter-human microdroplets.

The study also had limitations, such as that the collection of samples was carried out in the months of November and December, a period in which the number of new cases was less than 1,000 in Lima, Peru, according to the National Center for Epidemiology, Prevention and Control of Diseases. It is important to indicate that the WHO mentions that the biological basis for the possibility of infection through contact with surfaces and food is related to fomites, which is still considered a concept with contradictory evidence (40). Furthermore, the study was carried out only in districts of Lima, Peru, and not in other regions of the country; however, it should be considered that these districts had the highest number of infections in the most critical phase of the pandemic in Peru.

Another limitation is the low virus isolation in samples with higher Ct value, Ct >31 by RT-PCR method. The RT-PCR Ct value correlates strongly with culturable virus and likelihood of infectivity. In this sense, a higher number of positive samples provides us with the probability of a greater number of virus isolations (8). Another aspect to consider is that the surfaces under investigation were frequently cleaned during the study period, which could affect the possibility of positive results, but would not eliminate virus detection, as described in previous research (29).

Peru has been one of the countries most affected by the COVID-19 pandemic, and although it adopted early measures of social immobilization, some essential activities were maintained, such as the provision of food in markets, supermarkets, banking services, and public transportation, places with conditions for direct and indirect infection with SARS-CoV-2. The results of the study showed the presence of SARS-CoV-2 only on the surface of ATMs, which is consistent with the current literature that reports specific findings of this virus on various surfaces. It should be noted that the fact that SARS-CoV-2 was not detected on most surfaces does not in any way imply that measures such as surfaces and food disinfection should be suspended, since these public health strategies not only increase the probability of reducing the viral transmission (regardless of its magnitude) (40) but, more importantly, help to break the transmission chain of other pathogens, such as enteropathogenic bacteria, which are also a problem in Latin American health systems (28).

In conclusion, the frequency of disinfection measures should be maintained and increased, despite the negative results. New studies should be developed in periods of high contagion in the community and refrigerated and frozen foods should be assessed, as well as new variants of COVID-19 that are circulating in our country.

## Supplementary Material

Click to view [pdf].
